# Activation of GPR40 attenuates chronic inflammation induced impact on pancreatic β-cells health and function

**DOI:** 10.1186/1471-2121-15-24

**Published:** 2014-06-30

**Authors:** Mahesh Kumar Verma, Manoj Kumar Sadasivuni, Aggunda N Yateesh, Korrapati Neelima, Srikanth Mrudula, Madhusudhan Reddy, Rachapalli Smitha, Sanghamitra Biswas, Bhawna Chandravanshi, Puttrevana M Pallavi, Anup M Oommen, Madanahalli R Jagannath, Baggavalli B Somesh

**Affiliations:** 1 Connexios Life Sciences Pvt Ltd, Bangalore, India

**Keywords:** GPR40, β-cell apoptosis, β-cell survival, Inflammation, cAMP, ATP, Ca^+2^, Insulin content, Insulin secretion

## Abstract

**Background:**

Chronic inflammation-mediated β-cell apoptosis is known to decrease β-cell mass in diabetes leading to reduced insulin secretion. Exposure to pro-inflammatory cytokines can stimulate apoptosis in pancreatic β-cells. The G protein coupled receptor 40 (GPR40) is implicated for glucose induced insulin secretion. We hypothesized that GPR40 activation can protect β-cells from inflammation-induced apoptosis and restore glucose stimulated insulin secretion.

**Results:**

By exposing NIT1 insulinoma cells and rat islets to a cocktail of pro-inflammatory cytokines (TNFα and IL1β), we mimicked inflammatory signaling as seen by JNK and NFκB activation and increased mRNA levels of TNFα, IL1β and NOS2a. These changes were reversed by pharmacological activation of GPR40 by a specific, small molecule, CNX-011-67. Further, GPR40 activation reduced inflammation-mediated oxidative and endoplasmic reticulum (ER) stresses. Importantly, GPR40 activation decreased inflammation-induced apoptosis as measured by key markers. These impacts of GPR40 were mediated through activation of PLC, CaMKII, calcineurin and cAMP. Cell survival was also enhanced by GPR40 activation as seen from the increased phosphorylation of Akt/PKB and enhanced expression of BCL2 and PDX1 genes. Interestingly, GPR40 activation restored both, inflammation-mediated inhibition on insulin secretion and intracellular insulin content.

**Conclusions:**

In this study, we provide evidences that CNX-011-67, a GPR40 agonist, reduces inflammatory signaling and apoptosis in pancreatic β-cells while promoting insulin secretion and synthesis. Activation of GPR40 leads to attenuation of β-cell dysfunction caused by chronic inflammation and thus could be of immense clinical value to improve insulin secretion and β-cell survival.

## Background

Reduction in absolute β-cell mass and/or function is linked to type-1 and type-2 diabetes mellitus [1-3]. This decrease in β-cell mass is primarily due to apoptosis driven by an increase in cytokine levels and/or a nutrient overload [4,5]. In fact, nutrient overload as represented by elevated levels of saturated fatty acid and glucose increases cytokine production from β-cells, activates JNK and NFκB signaling pathways [6], and triggers oxidative and ER stresses [7-11]. These conditions (nutrient overload) result in chronic low grade inflammation [12-14] which play a central role in β-cell apoptosis [15]. Hence, it becomes imperative to delineate impacts of this chronic low grade inflammation and to make efforts overcome underlying severe consequences.

The β-cell mass is positively regulated by multiple mechanisms [16] and mild hyperglycemia itself can yield a compensatory increase in mass since glucose regulates β-cell growth [17]. Insulin secreted from β-cells in response to glucose activates Akt/PKB, a known inducer of cell growth and survival through the IRS-PI3K pathway. Similarly, IGF, insulin receptor, IRS2 and Akt have been associated with β-cell growth [18-23]. Thus, activation of insulin signaling by stimulating its secretion can positively impact β-cell survival and growth. For instance, GLP1 treatment, which enhances insulin secretion, has a positive impact on β-cell mass [24-27], and an increase in cAMP levels as mediated by GLP1 has been shown to reduce inflammatory signaling and apoptosis in β-cells [28-30].

The G-protein coupled receptor 40 (GPR40), also known as free fatty acid receptor 1 (FFAR1) is highly expressed in pancreatic β-cells. It is involved in fatty acid mediated potentiation of insulin secretion. Activation of GPR40 can increase insulin secretion [31-34] through phopsholipase-C (PLC) -Ca^+2^ pathway only at stimulatory glucose concentration.

Here, we propose that an increase in insulin secretion mediated by GPR40 can activate Akt/PKB to positively impact β-cell survival. Activation of GPR40 is also known to improve calcium flux in β-cells under chronic inflammatory conditions [13]. Since calcium dynamics and cAMP levels are coupled [35], we propose that GPR40 activation also attenuates inflammatory signaling.

In this study, we activated GPR40 using a specific small molecule agonist (CNX-011-67). Pharmacological activation of GPR40 by CNX-011-67 was very specific as an increase in cytoplasmic calcium flux was seen only in cells expressing GPR40 and no calcium flux was observed in cells which did not express GPR40. Moreover, CNX-011-67 mediated calcium flux was mediated through PLC pathway. Treatment of NIT1 cells and rat islets with CNX-011-67 showed a reduction in inflammatory signaling, inflammatory cytokines gene expression, cellular oxidative and ER stresses while increasing insulin secretion, intracellular insulin content and pro-survival signaling pathways. Taken together, GPR40 agonists provide a novel tool to counteract inflammation mediated β-cell dysfunction.

## Results

### Pharmacological activation of GPR40 enhances cytoplasmic calcium level in PLC dependent manner

**Figure 1:**
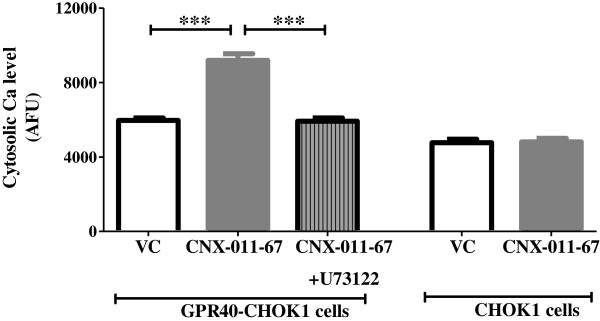
**CNX-011-67 is a specific agonist of GPR40.** CNX-011-67 induced GPR40 activation was measured in GPR40 over-expressing CHOK1 cells by its potency to increase in cytoplasmic calcium level which was abolished by PLC inhibitor (U73122) (n = 4, ***P < 0.001). CNX-011-67 did not increase any cytoplasmic calcium level in cells not expressing GPR40.

GPR40 activation by small molecule agonist (CNX-011-67, 1 μM) significantly increased cytoplasmic calcium level (9187 AFU versus (vs) 5967 AFU of control) only in CHOK1 cells over-expressing mouse GPR40 compared to the control (Figure 1). The cytoplasmic flux induced by CNX-011-67 was PLC-dependent as treatment with a PLC inhibitor (U73122) abolished this flux (5921 AFU vs 9187 AFU of CNX-011-67; Figure 1). Normal CHOK1 cells which did not express GPR40 showed no increase in cytoplasmic calcium in response to CNX-011-67 (4813 AFU vs 4768 AFU of control, not significant). This data demonstrates that CNX-011-67 specifically activates GPR40.

### Activation of GPR40 attenuates cellular inflammation

**Figure 2:**
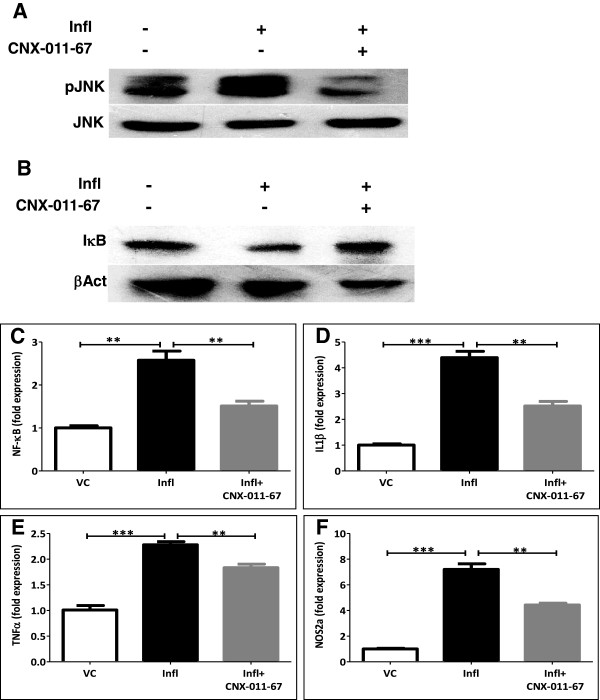
**GPR40 activation by CNX-011-67 reduces cellular inflammation.** Western blot of NIT1 cells treated with inflammatory cytokines in presence or absence of GPR40 agonist **(A, B)**. Inflammatory cytokines (TNFα + IL1β) increased JNK phosphorylation **(A)** and reduced IκB level **(B)** in NIT1 cells which were reversed by GPR40 activation. Total JNK and β-actin were used as loading control for normalization. Expression of NF-κB **(C)**, IL1β **(D)**, TNFα **(E)** and NOS2a **(F)** genes were up-regulated under inflammatory conditions and were down-regulated by GPR40 activation in rat islets. Gene expression was measured by qRT-PCR. (n = 4, **P < 0.01, ***P < 0.001).

Under chronic inflammatory conditions, NIT1 cells showed a 4.4 fold increase in JNK phosphorylation (Figure 2A) and 60% decrease in IκB levels (Figure 2B) compare to untreated cells, indicative of increased NFκB signaling. Activation of GPR40 decreased inflammation-induced JNK phosphorylation (1.5 fold of control vs 4.4 fold with inflammation) and increased IκB levels (1.3 fold of control vs 0.4 fold with inflammation) in NIT1 cells (Figure 2A and B).Gene expression of both NF-κB (Figure 1C) and pro-inflammatory cytokines such as IL1β (Figure 1D), TNFα (Figure 1E), and NOS2a (Figure 1F) was up-regulated by 2.6, 4.3, 2.2 and 7.2 fold respectively under chronic inflammation in rat islets, consistent with increased JNK and NF-κB activation. GPR40 activation reduced expression levels of NF-κB and pro-inflammatory genes to 1.5 (NF-κB), 2.5 (IL1β), 1.8 (TNFα) and 4.4 fold (NOS2a) respectively. These results demonstrate that activation of GPR40 can negate inflammatory signaling in β-cells.

### GPR40 activation reduces cellular stress in β-cells

**Figure 3:**
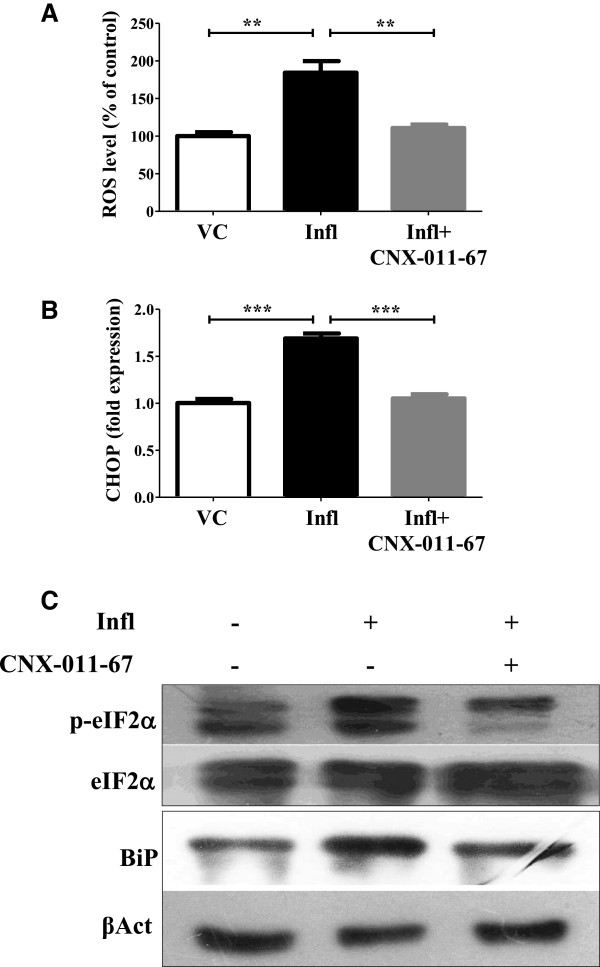
**Chronic GPR40 activation reverses inflammation mediated increase in oxidative and ER stress. (A)** NIT1 cells were treated for 72 h as indicated and ROS levels were measured by DCF fluorescence. ER stress was measured by expression of CHOP (DDIT3/GADD153) gene in rat islets cultured under inflammation with or without GPR40 activator **(B)**. (n = 4, **P < 0.01, ***P < 0.001). **(C)** NIT1 cells were treated as indicated and total cell lysates were used for western blotting against BiP (GRP78/HSPA5) and p-eIF2a to study ER stress. β-actin and total eIF2a were used as loading control for normalization.

Since increased cellular stress is a major contributing factor for β-cell apoptosis, we investigated whether GPR40 activation influences cellular stress (oxidative and ER stress). Under conditions mimicking chronic inflammation, NIT1 cells showed 1.8 fold increase of ROS compared to control which was brought back to normal upon GPR40 activation (Figure 3A). Similarly, chronic inflammation induced CHOP (DDIT3/GADD153) gene expression up to 1.6 fold in rat islets which was brought back to 0.6 fold upon GPR40 activation (Figure 3B). Like CHOP gene expression, inflammation conditions increased BiP level (1.4 fold of control) and eIF2α (1.2 fold of control) phosphorylation in NIT1 cells (Figure 3C) which were reversed upon GPR40 activation (0.6 and 0.7 fold of control respectively) (Figure 3C). Taken together, these data demonstrate that activation of GPR40 significantly lowers inflammatory signaling and cellular oxidative and ER stress.

### GPR40 activation protects β-cells from inflammation-induced apoptosis

**Figure 4:**
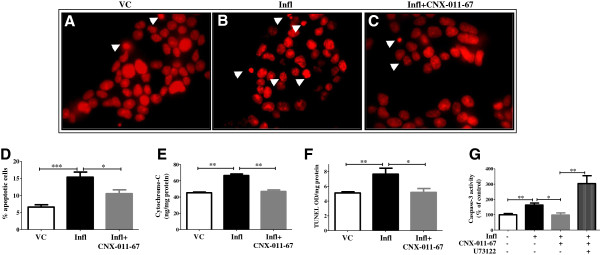
**Chronic GPR40 activation reverses inflammation mediated increase in apoptosis.** Apoptotic cells were visualized as condensed nuclei by propidium iodide fluorescence after chronic treatment of NIT1 cells with inflammatory cytokines in presence or ansence of GPR40 agonist **(A-D)**. Representative pictures showing condensed nuclei with white arrow **(A-C)** demonstrating an increased number of apoptotic cells under inflammation and reversal in apoptotic cells number by GPR40 activation. Thirty images having more than 800 total numbers of nuclei were counted and quantification data were represented by bar graph **(D)**. Inflammation conditions increased and GPR40 agonist decreased cytochrome-c level in rat islets as measured using ELISA assay **(E)**. In NIT1 cells, chronic inflammation increased and GPR40 agonist decreased TUNEL positivity **(F)**. Similarly, inflammation increased caspase-3 activity **(G)** in NIT1 cells which was reduced by GPR40 agonist. Caspase-3 activity was measured by its ability to cleave DEVD substrate and release of fluorescence. GPR40 induced inhibition of caspae-3 activity was abolished by incubating cells with PLC inhibitor (U73122). (n = 4, *P < 0.05, **P < 0.01, ***P < 0.001).

To support the results from cellular oxidative and ER stress, we next showed cellular apoptosis by different methods. Treatment with chronic inflammation caused nuclear fragmentation in NIT1 cells as measured by a significant increase in number of condensed nuclei (Figure 4A, B). Quantification data revealed that inflammation caused more than two fold increase in these condensed nuclei (15.4% apoptotic nuclei under inflammation compared to 6.6% in control; Figure 4D). GPR40 activation under this condition reduced nuclear fragmentation (Figure 4C) as number of condensed nuclei was decreased (10.5% apoptotic nuclei; Figure 4C, D). Chronic inflammation enhanced cytochrome-c levels (Figure 4E) with a concomitant increase in Terminal deoxynucleotidyl transferase dUTP nick end labeling (TUNEL) positivity (Figure 4F). Similarly, caspase-3 activity was also increased under chronic inflammation (163% of control; Figure 4G). GPR40 activation significantly decreased these apoptotic effects of inflammation as cytochrome-c level (Figure 4E), TUNEL positivity (Figure 4F) and caspase-3 activity were reduced (98% of control; Figure 4G). The GPR40-mediated inhibition of caspase-3 activity was PLC-dependent since treatment with PLC inhibitor (U73122) attenuated the effect of CNX-011-67 (Figure 4G). Interestingly, PLC inhibition increased caspase-3 activity more than chronic inflammation itself (Figure 4G). Thus, activation of GPR40 reduced inflammation-induced apoptosis of β-cells.

### GPR40 activation reduces apoptosis through CaMKII, Calcineurin and cAMP signaling

**Figure 5:**
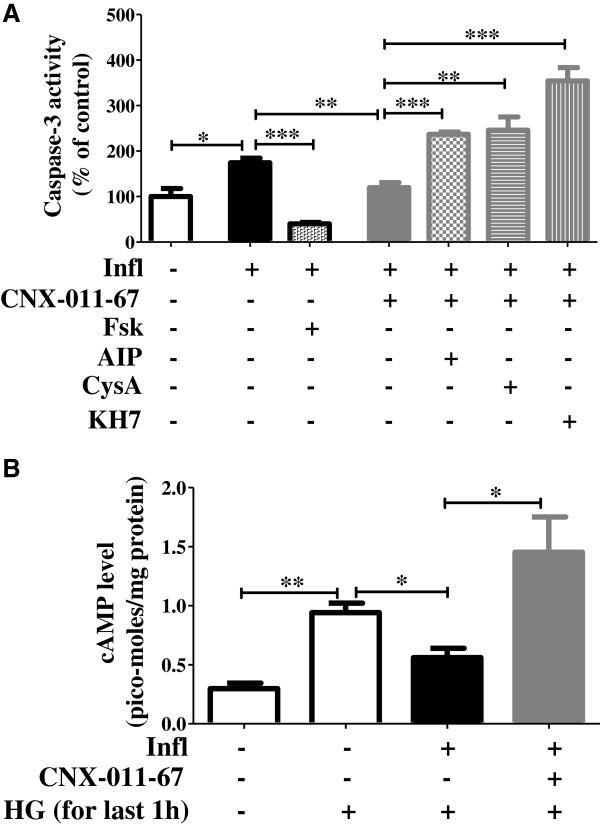
**Activation of GPR40 reduces apoptosis through cAMP and calcium signaling. (A)** NIT1 cells were cultured under inflammation conditions with or without GPR40 agonist for 72 h in presence of CaMKII (AIP), Calcineurin (CsA) and soluble ADCY (KH7) inhibitors or cAMP activator (Fsk) as indicated **(A)**. GPR40 agonist and Fsk reduced inflammation induced caspase-3 activity. AIP, CysA and KH7 blocked GPR40 agonist’s ability to reduced caspase-3 activity. **(B)** After chronic culture for 72 h, NIT1 cells were pre-treated with low glucose containing KRBH followed by treatment for 1 h with low or high glucose and cAMP levels in cells were determined by enzyme immunoassay. (n = 4, *P < 0.05, **P < 0.01, ***P < 0.001).

In order to decipher how GPR40 activation rescued β-cell apoptosis, we measured caspase-3 activity under chronic inflammation and used various pharmacological inhibitors to dissect out the underlying molecular mechanism. Since an increase in cytoplasmic calcium level activates CaMKII and calcineurin, we inhibited their activity using AIP and cyclosporine-A respectively [36,37] and measured changes in GPR40 mediated caspase-3 activity. Activation of GPR40 reduced inflammation-induced caspase-3 activity (174% of control under inflammation vs 120% under CNX-011-67) and this reduction was abolished by the inhibition of either CaMKII (237% of control) or calcineurin (246% of control) (Figure 5A). These data indicate an important role for GPR40-induced calcium flux in decreasing apoptosis. Interestingly, inhibition of either CaMKII or calcineurin increased caspase-3 activity significantly more than chronic inflammation alone (Figure 5A).

Increase in cytosolic calcium activates calcium-induced adenylate cyclase in pancreatic β-cells thereby increasing cAMP levels. Therefore, we assessed whether GPR40 activation increased cAMP in β-cells, which may help inhibit apoptosis. Under stimulatory glucose concentrations in β-cells, we found that cAMP levels were increased (0.94picomoles/mg protein at high glucose vs 0.30picomoles/mg protein at low glucose) (Figure 5B). Under chronic inflammatory conditions, cAMP levels were significantly reduced even in the presence of stimulatory glucose concentrations (0.56picomoles/mg protein). This effect was restored by GPR40 activation (cAMP levels: 1.45picomoles/mg protein) (Figure 5B). To demonstrate the role of GPR40-induced cAMP on apoptosis, we inhibited a soluble isoform of ADCY with ADCY inhibitor (KH7) [38]. Similar to the data obtained after CaMKII and calcineurin inhibition, treatment with KH7 abolished the impact of GPR40 on caspase-3 activity (354% of control with KH7 vs 120% with CNX-011-67) (Figure 5A). Consistent with the results seen upon PLC, CaMKII and calcineurin inhibition; ADCY inhibitor increased caspase-3 activity in β-cells significantly more than with inflammation alone (Figures 4F and 5A), indicating their role for normal β-cell functions. To better understand the role of cAMP on β-cell apoptosis, we activated ADCYs to increase cAMP levels using forskolin under inflammatory conditions and measured its effect on apoptosis. As seen in Figure 5A, forskolin significantly reduced caspase-3 activity (40% of control vs 174% under inflammation) indicating a key role of cAMP to inhibit β-cells apoptosis. Taken together, our data demonstrate that activation of GPR40 reduces inflammation-dependent apoptosis through a mechanism involving PLC, CaMKII, calcineurin and cAMP.

### GPR40 activation enhances cell survival signaling

**Figure 6:**
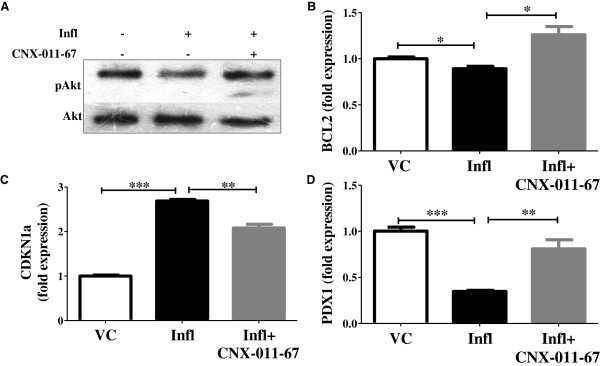
**GPR40 activation and β-cells survival. (A)** Phosphorylated Akt levels were measured by western blotting of NIT1 cells cultured for 72 h as indicated. Total Akt levels were measured for normalization. Chronic inflammation down-regulated expression of bcl2 **(B)** and pdx1 **(D)** whereas it up-regulated expression of cdkn1a **(C)** gene in rat islets. Activation of GPR40 partially reversed expression of these genes. Gene expression was quantified by qRT-PCR. (n = 4, *P < 0.05, **P < 0.01, ***P < 0.001).

We next examined weather GPR40 activation led to cell survival as yet another mechanism to counteract apoptosis. Activation of Akt/PKB, a known cell survival/growth signal [39], was reduced under chronic inflammatory conditions compared to control conditions (0.54 fold of control). GPR40 activation restored Akt/PKB phosphorylation under inflammatory conditions (1.51 fold of control vs 0.54 fold under inflammation) (Figure 6A). Expression of the anti-apoptotic gene BCL2, was decreased under chronic inflammatory conditions (0.89 fold of control). Activation of GPR40 increased BCL2 expression under these conditions (1.26 fold of control) (Figure 6B). Expression of CDKN1a/p21, a cell cycle inhibitor, was also increased under inflammatory conditions (2.7 fold of control), which was reduced by GPR40 activation (2.1 fold of control) (Figure 6C). Further, Pdx1 expression, which is required for β-cell maintenance, was reduced under inflammatory conditions (0.35 fold of control) and was increased upon GPR40 activation (0.81 fold of control) (Figure 6D). Thus, these data demonstrate that GPR40 activation improves β-cell survival and maintenance.

### GPR40 activation improves insulin synthesis and secretion

**Figure 7:**
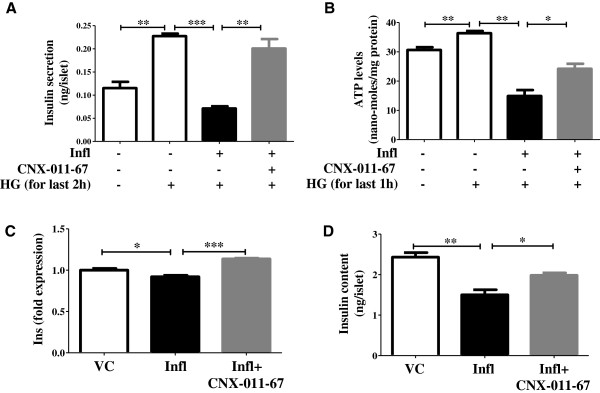
**GPR40 activation improves insulin synthesis and secretion. (A)** After chronic culture for 72 h, rat islets were pre-treated with low glucose containing KRBH followed by treatment for 2 h with low or high glucose. Amount of secreted insulin in buffer **(A)** and intracellular insulin content **(D)** were measured using ELISA. Likewise, NIT1 cells were also cultured and treated followed by cellular ATP levels estimation using luciferase based luminescence assay **(B)**. Insulin gene transcripts **(C)** were measured in rat islets after chronic culture under indicated condition by qRT-PCR. (n = 4, *P < 0.05, **P < 0.01, ***P < 0.001).

**Figure 8:**
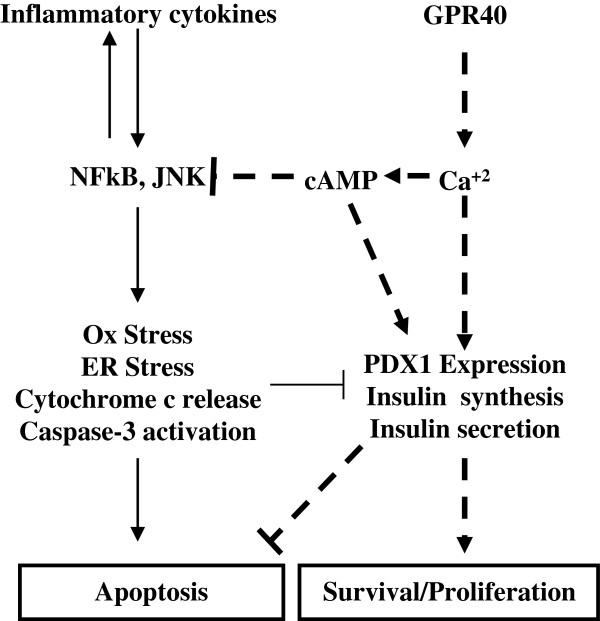
**Schematic representation of mechanisms involved for β-cells apoptosis caused by inflammatory cytokines exposure and its reversal by GPR40 activation.** Pointed arrows represent activatory signals and blunted lines represent inhibitory signals. Signals propagated from inflammatory cytokines are shown as normal arrows while signals propagated from GPR40 activation are shown as broken arrows/lines.

To verify that GPR40 activation plays a role in decreasing inflammation, we measured its impact on insulin secretion as an indicator of overall β-cell function. We observed impaired glucose-stimulated insulin secretion under chronic inflammatory conditions (0.07 ng/islet vs 0.22 ng/islet under control), which was significantly improved by GPR40 activation (0.20 ng/islet) (Figure 7A). GPR40 activation also led to an increase in insulin secretion from control islets (Additional file 1). Further, GPR40 activation increased cellular ATP (24nano-moles/mg protein), which is required for insulin secretion and was decreased under chronic inflammation (15nano-moles/mg protein vs 36nano-moles/mg protein under control condition) (Figure 7B). In keeping with these observations, we subsequently assessed the impact of GPR40 activation on insulin synthesis. Expression of insulin gene was significantly reduced under chronic inflammatory conditions (0.92 fold of control) (Figure 7C). Activation of GPR40 under these conditions increased insulin gene expression (1.14 fold of control) (Figure 7C). Consistent with the findings of insulin gene expression and insulin secretion, intracellular insulin content was decreased under chronic inflammatory conditions (1.5 ng/islet vs 2.4 ng/islet under control) and was restored by GPR40 activation (2.0 ng/islet) (Figure 7D).Based on our findings, we provide a schematic representation of how GPR40 activation led to counteract inflammation mediated deleterious effects on pancreatic β-cells (Figure 8). Exposure to inflammatory cytokines led to activation of NFκB and JNK which causes increased expression of pro-inflammatory cytokines, thus creating a positive feed back loop. NFκB and JNK activation lead to increase oxidative and ER stress, cytochrome-c release from mitochondria and Caspase-3 activation thereby causing apoptosis and reduction in insulin synthesis and secretion. Activation of GPR40 augments cytoplasmic calcium and cAMP levels which potentiate insulin synthesis and secretion thereby autocrine cell survival signaling. Moreover, elevated cAMP levels reduce activation of NFκB and JNK and hence block inflammatory signaling (Figure 8).

## Discussion

In this present study we showed that activation of GPR40 by a small molecule, CNX-011-67, rescued inflammation-mediated apoptosis in pancreatic β-cells. We demonstrated that chronic inflammation, which has been shown to severely impact β-cells’ function and mass [14], caused an augmentation in cellular oxidative and ER stress, expression of pro-inflammatory cytokines genes and apoptosis. We found that all these impacts of chronic inflammation were reversed by GPR40 activation. Also we have observed no significant change in the expression of GPR40 under inflammation condition with or without the receptor activation (data not shown).

Chronic inflammation has been a mechanism implicated for β-cell apoptosis in both type-1 and type-2 diabetes [4,40-42]. Hence, it is important to counteract inflammation-mediated changes in β-cells in order to protect them from apoptosis. Therefore, we evaluated the potential of GPR40 activation as a mechanism to neutralize inflammation mediated consequences. We have activated GPR40 using a small molecule agonist (CNX-011-67) which showed an increase in calcium flux. CNX-011-67 is a specific GPR40 agonist as it enhanced calcium flux only in cells expressing GPR40. Moreover, inhibition of PLC reversed CNX-011-67 induced calcium flux as GPR40 activation mediates its effect via PLC-Ca^2+^ pathway [31].

Since, inflammatory cytokines exert their effects through activation of JNK and NFκB, we have showed that treatment with GPR40 agonist was able to reduce their activation. These findings were also supported by a decrease in pro-inflammatory cytokines gene expression by GPR40 agonist. Activation of JNK and NFκB has been shown to up-regulate these pro-inflammatory cytokines [43], hence their reduced activation by GPR40 agonist led to a decreased expression of these genes (TNFα, IL1β, Nos2a). Thus, GPR40 activation not only blocked impact of those inflammatory cytokines added to culture medium but also inhibited their production from β-cells thereby plunging chronic low grade inflammation maintenance.

Elevated cellular oxidative and ER stress under nutrient overload condition can cause β-cells apoptosis [7-11]. In fact, we also observed increased oxidative and ER stress after pro-inflammatory cytokines treatment which was abolished by GPR40 agonist. Under inflammatory conditions, an increased level of β-cell death was observed as measured by cytochrome-c release, TUNEL positivity, Caspase-3 activation and nuclear fragmentation. In consistent with above mentioned findings with GPR40 activation, we observed diminished apoptosis in pancreatic β-cells upon GPR40 agonist treatment.

In order to dissect the mechanism by which GPR40 activation reduced β-cells apoptosis, we used various pharmacological agents to inhibit or activate key intracellular signaling pathways. As expected, inhibition of PLC signaling by U73122 abolished GPR40 agonist impact on apoptosis because GPR40 exerts its effect by PLC activation. Interestingly, PLC inhibition caused a higher increase in Caspase-3 activity compared to inflammation alone, indicating its importance for normal physiological functions of β-cells. In fact, elevated glucose level can cause activation of PLC signaling in islets [44]. Activation of PLC leads to ER calcium release; hence we next tested the role of calcium signaling for GPR40 induced reduction in apoptosis. Increase in cytoplasmic calcium can lead to activation of calcium-calmodulin dependent protein kinase (CaMK) and calcineurin-NFAT pathways. In order to specify which of these pathways was involved in the ability of GPR40 to counteract inflammation induced apoptosis, we inhibited each of these and observed their effect. Inhibition of CaMKII, the major isoform present in β-cells, showed a pronounced increase in caspase-3 activity. Similar results were obtained after calcineurin inhibition. Interestingly, inhibition of either CaMKII or calcineurin caused increased caspase-3 activity higher than that achieved by inflammation itself, signifying their importance in normal physiology of β-cells.

Increase in cytoplasmic calcium level is coupled with an increase in cAMP level due to presence of calcium activated ADCY in β-cells. We tested the possibility that increase in cytoplasmic calcium level by GPR40 activation would lead to a corresponding increase in cAMP level. We found that cAMP levels were decreased under inflammation condition and were restored by GPR40 agonist. To demonstrate the importance of GPR40-induced cAMP level for caspase-3 activity, we inhibited a soluble isoform of ADCY present in β-cells which is known to mediate cAMP oscillation [38,45]. Similar to data obtained with PLC, CaMKII and calcineurin inhibitor; inhibition of sADCY caused a drastic increase in caspase-3 activity at levels higher than that achieved with inflammation alone. Since these inhibitors are known to suppress insulin secretion, their exposure might have reduced autocrine survival ‘insulin-PI3K-Akt’ signaling. In contrast, increase in cAMP level by forskolin led to a significant reduction in caspase-3 activity indicating an independent role of cAMP for reduction in β-cell death. Treatment with GLP1, which can also increase cAMP levels, has been shown to reduce apoptosis in pancreatic β-cells [28-30]. Thus, our data collectively demonstrate that GPR40 activation relayed its signal to multiple arms of intracellular signaling pathways to reduce inflammation mediated apoptosis.

GPR40 activation not only inhibited β-cell apoptosis but also activated cell survival pathways. Its activation led to an increase in ATP level which together with elevated Ca^2+^ and cAMP level caused a significant increase in glucose-stimulated insulin secretion. This secreted insulin can lead to Akt/PKB phosphorylation in an autocrine manner. The level of phosphorylated Akt, a well known marker for cell survival and proliferation, was reduced under chronic inflammation conditions, together with a reduction in insulin secretion. GPR40 agonist reversed this decrease in pAkt level. Similarly, BCL2 and PDX1 expression were reduced under inflammation and were restored by GPR40 agonist. PDX1 maintains β-cell phenotype and also regulates insulin synthesis. In consistent with PDX1 data, inflammation reduced insulin gene transcription and intracellular insulin content and their levels were restored by GPR40 agonist. Under this condition we did not observe any change in the glucagon expression in rat islets (data not shown). This is consistence with the fact that GPR40 expression in predominant only in β-cells.

We have earlier showed that activation of GPR40 by oral administration of CNX-011-67 in male ZDF rats reduces β-cells apoptosis, increases insulin and PDX1 positive cell number and insulin secretion [46]. It should be noted that ZDF rat is an animal model of diabetes having high chronic systemic inflammation [47,48]. Hence, our *in vitro* data are in agreement with *in vivo* findings. Taken together, our data demonstrate that GPR40 activation by CNX-011-67 reduces inflammation induced apoptosis, enhances β-cell survival and improves β-cell function as measured by insulin synthesis and secretion.

## Conclusions

In this study, we demonstrated that activation of GPR40, which is implicated for glucose induced insulin secretion, can rescue pancreatic β-cells from inflammation induced dysfunction. GPR40 activation increased cytoplasmic calcium level in a PLC dependent manner. We also established the molecular link of GPR40 activation and downstream calcium flux to cellular cAMP levels. GPR40 mediated its impact through CaMKII, NFAT and cAMP as their inhibition totally reversed the protective impact on β-cells apoptosis. Moreover, GPR40 activation promoted β-cell survival signaling which was impaired under chronic inflammatory conditions. These survival signaling might have been initiated by insulin as GPR40 activation led to enhanced insulin secretion. This study provides basis for the development of GPR40 activators that might be an effective therapeutic strategy to combat β-cells dysfunction caused by chronic inflammation.

## Methods

### Rat islet isolation

Male Wistar rats (8–10 weeks, 180-240gm body weight; Charles River Lab, USA) were used for islet isolation. All experimental protocols have been approved by Institutional Animal Ethics Committee (IAEC) of Connexios Life Sciences, which is recognized by the Committee for the Purpose of Control and Supervision on Experiments on Animals (CPCSEA), India. Excess anesthesia was used to kill the animals and the pancreata were cut into 1-2 mm pieces in HBSS (pH7.4; Sigma) followed by digestion with collagenase-II (2 mg/ml in HBSS; Sigma) at 37°C for 20 min. The reaction was stopped by adding two volumes of culture medium (RPMI containing 10%FBS; Invitrogen) and gently triturated. The cell pellet was obtained after centrifugation at 100X*g* for 5 min and then washed resuspended in 4 ml of Histopaque (1.119 gm/ml; Sigma). Histopaque (1.077 mg/ml; 3 ml) followed by culture medium were overlaid on this suspension and density gradient centrifugation was carried out. The islets were recovered from the interface of culture medium and Histopaque 1.077 and then washed with HBSS. The purified islets were then handpicked under a stereo-zoom microscope (Nikon, Japan) and used for subsequent experiments.

### Cell culture and treatment

NIT1 cell line (ATCC) was cultured under either control or inflammation (TNFα + IL1β, both 10 ng/ml) conditions for 72 h in presence or absence of GPR40 agonist (CNX-011-67, 1 μM). For Caspase-3 assay, NIT1 cells were cultured for 72 h in presence or absence of the following pharmacological modulators- PLC inhibitor (U73122, 2 μM), soluble adenylate cyclase (ADCY) inhibitor (KH7, 30 μM), non-specific ADCY activator (forskolin, 10 μM), calcium-calmodulin kinase-II (CaMKII) inhibitor (AIP, 1 μM) and calcineurin inhibitor (Cyclosporin A: CysA, 1 μM). Treatments for rat islets were the same as described for NIT1 cells above.

### Calcium flux

CHOK1 cells over-expressing mouse GPR40 (Cytobox) were plated in 96-black well plate. Cells were washed with KRBH and loaded with Fluo-4-AM, a calcium indicator fluorescent dye (Invitrogen), at 37°C for 1 h. Basal fluorescence readings were taken at 485 nm excitation and at 520 nm emission. Cells were then induced with GPR40 agonist (CNX-011-67, 1 μM) in presence or absence of PLC inhibitor (U73122, 10 μM) followed by fluorescence readings for induced calcium flux. Changes in fluorescence readings between basal and induced conditions in different treatments were defined as arbitrary fluorescence units (AFU). Normal CHOK1 cells not expressing GPR40 were used as a control for the experiment.

### Western blotting

After incubation, NIT1 cells were lysed and total proteins were resolved by SDS-PAGE followed by transfer to nitrocellulose membrane. After blocking with BSA, the membrane was incubated overnight at 4°C with primary antibody followed by washing and incubation with HRP-conjugated secondary antibody. Primary antibodies used are pAKT, AKT, pJNK, JNK, IkB and β-actin (Cell Signaling Technology). The blot was developed using chemiluminescence substrate (Pierce) and exposed to X-ray film. Densitometric analyses were carried out using Image-J (NIH) software.

### RNA isolation, reverse transcription and quantitative real time polymerase chain reaction (qPCR)

Total RNA was extracted from treated islets using Trizol reagent (Sigma, St. Louis, MO, USA), and was used as a template for cDNA synthesis with reverse transcriptase and random hexamer primers (ABI, CA, USA). Quantification of gene expression was done using SYBR Green PCR Master Mix (Eurogenetic, Belgium) using the ABI7500 fast thermal cycler. We examined expression for those genes whose primers generated a single peak in the melting curve analysis and a single, specific band in agarose gel electrophoresis. Genes analyzed in this study were NF-κB, IL1β, TNFα, NOS2a, CHOP, BCL2, CDKN1A, PDX1 and Insulin. β-actin or 18S rRNA was used as a housekeeping gene control. The primer sequences are provided in the Additional file 2.

### Caspase-3 assay

After 72 h of incubation, NIT1 cells were lysed in lysis buffer followed by estimation of Caspase-3 activity. Caspase-3 activity was measured by cleavage of its substrate (Ac-DEVD-R110; Invitrogen) and release of fluorescent R110 which was measured at 485 nm excitation and 530 nm emission. Activity of caspase-3 (as nano-moles of R110 released) was normalized to total cellular protein. Change in Caspase-3 activity under different conditions was represented as% of control.

### Nuclear fragmentation assay

NIT1 cells were fixed after chronic treatments of inflammation and GPR40 agonist. Nuclei were stained with propidium iodide (PI) and morphology of nuclei was studied microscopically. Images were captured at 100X magnification for analysis. For each set more than 800 nuclei were counted and fragmented, shrunken or condensed nuclei were taken to indicate apoptotic nuclei. Data were presented as% of apoptotic nuclei.

### Cytochrome-c assay

Rat islets were treated under inflammation and GPR40 agonist. After treatment media was removed, islets were washed three times with PBS, followed by solubilization of the cell membrane using 1xPBS and 0.5% triton-X-100. Cytochrome C levels were measured according to the manufacturer’s instructions (RnD Biosystems).

### TUNEL assay

After chronic treatment with inflammation and GPR40 agonist, NIT1 cells were washed with PBS and fixed with 1% paraformaldihyde. Fragmented DNA was labeled using ApopTag® Peroxidase Apoptosis Detection Kit (Millipore) and peroxidase activity was measured using TMB-H_2_O_2_. TUNEL levels were normalized to total cellular protein amount.

### Insulin secretion and intra-cellular insulin content analysis

Following chronic treatment of inflammation and GPR40 agonist, size-matched islets were picked under a microscope and transferred into 24-well plates containing 1 ml KRBH buffer (2.5 mM glucose)/well and pre-incubated at 37°C/5% CO_2_ for 1 h. The buffer was then carefully removed from the wells and the islets were induced in fresh KRBH buffer (250 μl/well) containing low (2 mM) or high glucose (11 mM) at 37°C/5% CO_2_ for 2 h. The supernatant was removed from the islets and stored at −70°C for ELISA. Secreted insulin was measured in the KRBH buffer using ELISA (Mercodia) as per manufacturer’s instructions. Insulin secretion was represented as the amount of insulin secreted (ng) per islet. Islet lysates were used to measure intracellular insulin content, which was also represented as the amount of insulin (ng) per islet.

### Measurement of ATP and cAMP level

NIT1 cells were cultured under inflammation and GPR40 agonist and then washed with KRBH buffer followed by pre-incubation at 37°C/5% CO_2_ for 1 h in the same buffer. Subsequently, cells were treated with low (2 mM) or high glucose (11 mM) at 37°C/5% CO_2_ for 1 h followed by estimation on cAMP or ATP in cell lysates. ATP determination kit (Invitrogen) and Amersham cAMP Biotrak Enzymeimmunoassay (EIA) System (GE Healthcare) were used for the estimation of ATP and cAMP respectively.

### Measurement of cellular ROS level

After chronic treatments of inflammation and GPR40 agonist for 72 h, NIT1 cells were loaded for 1 h with DCFH-DA dye for ROS measurement (Invitrogen). Cells were lysed and lysates were transferred to 96-black well plate to measure increase in DCF fluorescence at 485 nm excitation and 528 nm emission. Amount of ROS was normalized to total cellular DNA which was measured using bis-benzamide fluorescence at 360 nm exication and 460 nm emission.

### Statistical analysis

Data are expressed as mean ± SEM and significance was calculated using the unpaired Student’s t-test. *indicates p < 0.05; **indicates p < 0.01; ***indicates p < 0.001 compared to the respective control. Each data point consists of four individual replicates and experiments were repeated to check reproducibility. Miscrosoft Excel was used for statistical analysis.

## Competing interest

The authors do not declare any competing interest.

## Authors’ contribution

VMK, SMK, YAN, NK, MS, MR, SR, SB, BC and PPM carried out experiments; VMK, SMK, SBP, MOA and JMR planned/executed the study and analyzed data. VMK, SMK, SBP wrote the manuscript. All authors read and approved the final manuscript.

## Supplementary Material

Additional file 1 Impact of GPR40 agonist (CNX-011-67) on insulin secretion from rat islets. Click here for file

Additional file 2 List of primers used in the study. Click here for file
