# Supplementary material for: Activation of GPR40 attenuates chronic inflammation induced impact on pancreatic β-cells health and function
Source: BMC Cell Biol. 2014 Jun 30;15:24. doi: 10.1186/1471-2121-15-24 (PMC4083038; doi:10.1186/1471-2121-15-24)
Supplement: Additional file 1 — Impact of GPR40 agonist (CNX-011-67) on insulin secretion from rat islets. [file 1471-2121-15-24-S1.doc]

**Additional File**

**Additional file-1. Impact of GPR40 agonist (CNX-011-67) on insulin secretion from rat islets.**

Rat islets were incubated under low glucose (LG, 2mM) or high glucose (HG, 11mM) or high glucose containing 1uM GPR40 agonist (CNX-011-67) for 2h. Amount of insulin secreted in buffer was measured using insulin ELISA kit. (n=4, *P<0.05, **P<0.01).
