# Supplementary material for: Activation of GPR40 attenuates chronic inflammation induced impact on pancreatic β-cells health and function
Source: BMC Cell Biol. 2014 Jun 30;15:24. doi: 10.1186/1471-2121-15-24 (PMC4083038; doi:10.1186/1471-2121-15-24)
Supplement: Additional file 2 — List of primers used in the study. [file 1471-2121-15-24-S2.doc]

**Additional File**

**Additional file 2:** List of primers used in the study

| **Gene Name** | **Forward Primer (5’ to 3’)** | **Reverse Primer (5’ to 3’)** |
| --- | --- | --- |
| NF-κB | ccatcctgaaggctaccaactaca | atcagcacccaaggacaccaaa |
| IL1β | tgacccatgtgagctgaaag | gggattttgtcgttgcttgt |
| TNFα | tgacccccattactctgacc | ggccactacttcagcgtctc |
| NOS2a | ggaagaaatgcaggagatgg | tctgcaggatgtcttgaacg |
| CHOP | gtctctgcctttcgcctttg | ggtgcccccaatttcatct |
| BCL2 | ggcagcagtgaagcaagcgc | gctctgatatgctgtccctggggt |
| CDKN1a | tcagtggaccagaagggaac | ctcgtcaccaaggacctacc |
| PDX1 | aacgccacacacaaggagaaca | aggaaagagtcccagaggcaga |
| Ins | ttcttctacacacacccaagtcccgt | actgatccacaatgccacgct |
| β-actin | gaaaatctggcaccacacct | agaggcgtacagggatagca |
| 18S rRNA | ggacacggacaggattgaca | cgctccaccaactaagaacg |
